# Intake Fraction of PM_10_ from Coal Mine Emissions in the North of Colombia

**DOI:** 10.1155/2018/8532463

**Published:** 2018-07-29

**Authors:** Heli A. Arregocés, Roberto Rojano, Luis Angulo, Gloria Restrepo

**Affiliations:** ^1^Grupo de Investigación GISA, Facultad de Ingeniería, Universidad de La Guajira, Riohacha, Colombia; ^2^Grupo Procesos Fisicoquímicos Aplicados, Facultad de Ingeniería, Universidad de Antioquia, Medellín, Colombia

## Abstract

Intake fraction was determined in this study to provide insight into population exposures to PM_10_ that is effectively inhaled due to emissions of an opencast coal mine. We applied the CALPUFF model to a coal mine in Northern Colombia, which has 6 active pits with an annual production of 33.7 million tons. We estimated the intake fractions for 7 towns through the integration of dispersion model results over the population data. The resulting average intake fractions were between 6.13 × 10^−9^ and 3.66 × 10^−8^ for PM_10_. 62.4% of the intake fractions in the domain were calculated within a 23 km radius from the coal mine and coved 44.3% of the total population in this area. We calculated an estimate point for morbidity impacts using standard epidemiological assumptions. It is estimated that there were annually 105835 restricted activity days and 336832 respiratory symptom cases due to the direct impact of the opencast coal mining. These data also provide a framework for improved understanding of the effect of coal mining in Colombia.

## 1. Introduction

Numerous epidemiological studies have found a strong association between exposure to particulate matter and adverse health effects [1–6]. Air pollution was estimated to cause 4.2 million premature deaths worldwide per year; this mortality is due to exposure to small particulate matter which causes cardiovascular and respiratory disease and cancers [7].

Approximately, 92% of the annual production of bituminous coal in Colombia is carried out in the northern part of the country by the opencast method, between the departments of Cesar and La Guajira, constituting an important zone of economic activities [8]. Open-pit mining is considered to be one of the main sources of particulate matter with aerodynamic diameter <10 *µ*m (PM_10_). The available evidence indicates that there are negative health impacts for people living in communities near coal mines [9–11]. Although numerous coal mining activities have been operating for more than 20 years in the north of Colombia, their direct impacts have not yet been determined. According to the most recent Colombian Air Quality Report [12], this area has the highest air pollution levels in Colombia. In addition, this zone has been most clearly identified as a public health concern by local residents. The same study shows the occurrences of events whose concentrations reached the categories “Damage to health for sensitive groups” and “Damage to health,” which deserves special attention that results in efforts focused at the local level to improve the state of quality of air. It is therefore essential that the long-term relative contribution of emissions from open coal mining sources to the total atmospheric PM_10_ concentrations is determined in order to establish their impacts and to develop suitable mitigation strategies. Due to the comprehensive amount of information needed to establish the impact of emissions from sources on population health, it is crucial to construct air pollution prediction models in order to develop effective control strategies and to estimate the cost benefits.

Intake fractions (iFs) relate pollutant emission sources to exposure to the pollutant [13]. iFs have been used in numerous studies to determine the adverse impacts of different pollutants on human health for various populations [13–15]. iF (also referred to as exposure efficiency or dose fraction) is a unitless value defined as the fraction of a pollutant or its precursor emitted from a source that is eventually inhaled by someone. The iFs are not a feature of the contaminant and are dependent on many factors like concentrations of pollutants released from the source, the distance between the point of emission and the exposed individuals, meteorological conditions, number of exposed individuals, and the time of exposure for these individuals. The iFs are usually estimated with atmospheric dispersion models that assess the levels of pollutant concentration in all receptors on the population assigned to each receptor or grid of domain [16]. To summarize the results of the model in a way that is directly applicable to the estimation of health effects, the concept of iFs is applied. When iFs are determined according to Bennett et al. [13] to estimate the health risks, the implicit assumption is that the health effect has a linear dose-response for the complete range of background pollutant concentrations in the affected region. However, the concept of determination of iFs is not only based on these assumptions, but may be modified according to evidence in health [16, 17].

In this article, we report iFs for 7 towns in close proximity to an opencast coal mine in relation to PM_10_ emissions from this mine with the CALPUFF model in order to establish the impacts on health. The main objectives were (i) to estimate the spatial variations of iFs for each town and (ii) to assess that annual impact on health according to iFs.

## 2. Methodology

For determining the PM_10_ intake fraction from coal mine emissions in the north of Colombia, the PM_10_ discharge into the atmosphere for 6 active pits with an annual production of 33.7 million tons was calculated.

**Figure 1:**
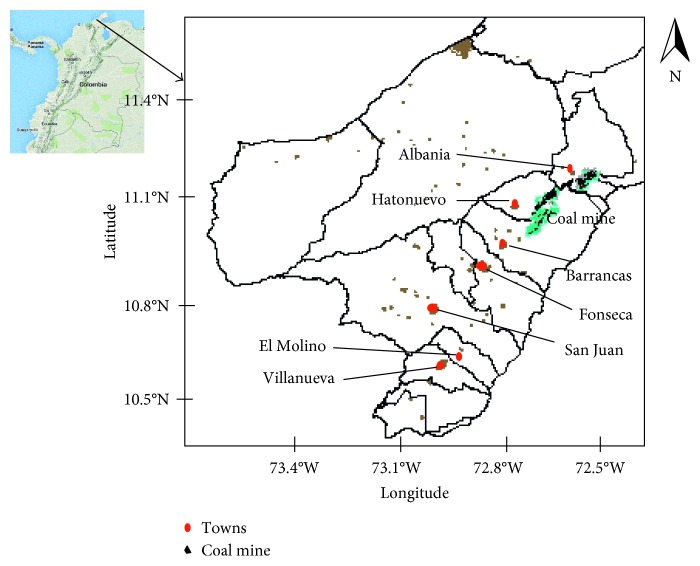
Towns and coal mine located in Northern Colombia. Points in red color represent the most populated towns in the region.

The modeling domain, using CALPUFF, was 150 km × 150 km with a resolution of 1 km^2^ (Figure 1). Our CALPUFF modeling methodology is described in depth by Arregocés et al. [18]. Briefly, given available surface data for 2014 and upper-air data were taken from the Almirante Padilla Airport. Surface observations were taken from meteorological stations of the National Climatic Data Center and the mining company, providing hourly observations from 6 stations across the domain area.

This approach of risk analysis by emission unit is limited to activities of the opencast coal mine and it does not consider other sources that can increase chances of the risk; it can ultimately sustain a more comprehensive risk assessment. The emissions were calculated using the emission factors from EPA for mine activities such as management of topsoil-coal, wind erosion, maintenance of roads, and storage of coal piles. All emissions were assumed to be uniform during the modeling period; this assumption is attributable to the limitations of the data. On the other hand, the iFs for 7 towns were calculated by the following equation:

(1) $$\begin{array}{c} \text{iFs}=\frac{\sum_{t=1}^{N}\;P_{i}\times \text{BR}\times C_{i}}{Q}, \end{array}$$

where the modeling domain was divided into 150 × 150 grid cells and *i* indexes these cells. In this calculation, *P*_*i*_ is the population in cell *i*, derived using 2005 projection population data, *C*_*i*_ is the incremental concentration at location *i* (g·m^−3^), BR is the population-average breathing rate (m^3^·s^−1^), for which we assume a nominal value of 20 m^3^·d^−1^, and *Q* is the emission rate of the pollutant. This intake fraction formula (and the values reported in this paper) is only directly relevant for human health risk assessment if risk is proportional to ambient concentration—without any strong nonlinearities or thresholds [19]. PM_10_ data were collected every two days from five stations of the open-pit mine monitoring network for the calibrated dispersion model over the annual period.

If the health effects caused by the pollutants have a linear dose-response function with no threshold above annual ambient concentrations established by the environmental authority or dose rate dependence, a straightforward calculation of iFs based on annual average concentrations directly corresponds to health benefit estimates. The analytical approach used for the annual health effect estimation of air pollution followed the subsequent main steps: (i) determination of PM_10_ concentrations in each 1 km^2^ grid in the domain using the CALPUFF model and (ii) estimation of the health effects of air pollution based on epidemiological techniques according to Ostro [20] and Abbey et al. [21]. Based on this technique, a population density proportional to each grid is assumed. The analysis reported here uses a similar approach to estimate the health effects of PM_10_ due to coal mine emissions in the north of Colombia. Dose-response functions that relate various health outcomes to air pollution are taken from the available previous research. Estimates of selected health effects of PM_10_ are generated by applying these functions to the PM_10_ concentration levels estimated from dispersion model. The dose-response functions allow determining the number of hospital respiratory disease admissions, emergency room visits, restricted activity days, and respiratory symptom cases. The estimated health impact can be represented as follows:

(2) $$\begin{array}{c} dH_{i}=b\times P_{i}\times dC_{i}, \end{array}$$

where *dH*_*i*_ is change in population risk of health effect *i*, *b* is slope from dose-response curve, *P*_*i*_ is population at risk of health effect in cell *i*, and *dC*_*i*_ represents the change in air pollution under consideration. The *b* expression involves calculating the partial derivate or slope of the dose-response functions, to provide an estimate of the change in the prevalence of a given health effect associated with change of PM_10_ concentrations. Sufficient information is provided by scientific research that allows selected dose-response functions [5, 14, 20, 22–24]. The *P*_*i*_ expression is the relevant population that is believed to be exposed and susceptible to the PM_10_ effect, and this may include the entire exposed population. Finally, the *dC*_*i*_ expression considers the change from the current PM_10_ levels due to emission source versus air local quality standard.

## 3. Results and Discussion

Inside the mine, there are 6 area sources of emissions (dump, pit, backfilling, pit development, coal piles, and unpaved road). The main source of emissions in the region is the opencast coal mine (Figure 1 and Table 1). No other economic activities in the region could be considered as relevant sources of emissions. The PM_10_ emissions during the transportation of material over unpaved roads within the mine are significant, and more than 10 tons per day are introduced into the atmosphere by this source in the region. Emissions from dumps are of greater concern since they are located at the highest point of the mine and therefore have the potential of being transported by wind action to very long distances outside of the mine. Even though emissions from pits are important in quantity, a high percentage of them remain within the pit due to air recirculation within the mine [25].

Few studies have examined the regional-scale variation of iFs for PM_10_ associated with opencast coal mining emissions. The majority of previous studies have examined the iFs for PM, for power plant emissions [19, 26, 27], traffic emissions [17, 28–31], industrial emissions [32, 33], and miscellaneous sources [34]. In Table 2, the PM_10_ iFs for the seven towns within close proximity of the opencast coal mine are presented. It is evident that the iFs ranges between 6.13 × 10^−9^ and 3.66 × 10^−8^, and these values are higher than those determined in another research [31, 35, 36]. The results show averages of daily iFs in the order of 10^−8^ for the 4 closest towns located in the open-pit coal mine, and the iFs for Barrancas town were the highest (3.66 × 10^−8^) thanks to high population density and the proximity to the mining emission areas. The iFs calculated for coal mines in our domain are significantly greater than the values from the other studies; these values are in consideration of multiples sources, size and resolution of the domain, population, and size of particulate matter. Zhou et al. [27] evaluated the influence of emission source location on population exposure to fine particles in China. They used modeling domain of 3360 by 3360 km (resolution of 28 km) that covered all the heavily populated areas in China with an approximate population density of 132 people km^−2^. On the other hand, Tainio et al. [34] evaluated the iFs in the European population for emissions of anthropogenic primary fine particulate matter (PM_2.5_) from emissions calculated for 205 point sources with detailed plant and stack characteristics, and area sources with aggregation to 112 source categories and 15 fuels; the population dataset contained data for 39 European countries. In comparison with the above studies, our modeling domain and population density are significantly less for explaining the results obtained.

The mean value for iFs in Albania, the closest town to an opencast coal mine, is 3.53 × 10^−8^, which is approximately a factor of 3.6 and 3.1, and is greater than the iF mean of El Molino town and Villanueva town, which are located more than 50 km from each other, respectively. The particulate matter can travel long distances in the atmosphere and cause a wide range of diseases and a significant reduction of life expectancy in most of the population. Greco et al. [29] investigated the spatial patterns of source particulate matter emissions to exposure. The results shows that half of the total exposure was reached by a median distance of 150 km from the originated source emissions, though this spatial extent varied across the different areas of domain studies.

**Figure 2:**
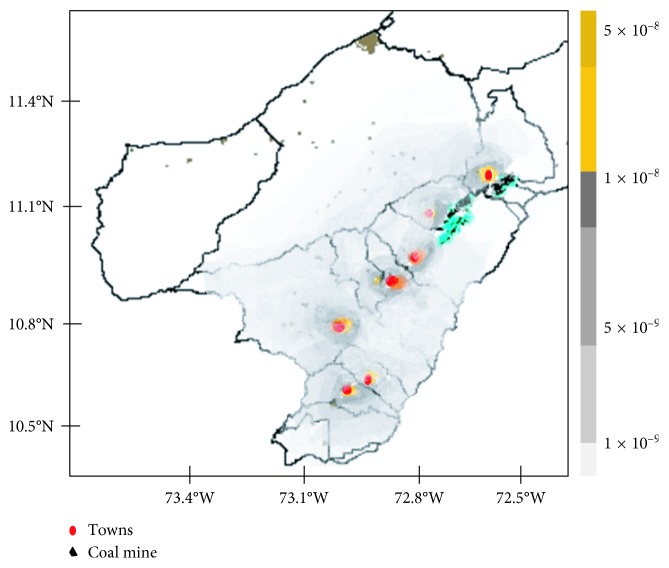
Map of regional-scale mining source intake fractions (PM_10_) for Northern Colombia.

The analysis of the PM_10_ spatial distribution shows that, in a radius of 23 km from the mining sources, the annual average PM_10_ increase associated with the opencast coal mine is 19.01 *µ*g·m^−3^; in this radius, the towns of Albania, Hatonuevo, and Barrancas are directly affected. In addition, in a radius greater than 50 km, the annual concentrations of PM_10_ increase associated with the mining sources is 3.52 *µ*g·m^−3^. The bigger the radius is, the lower their annual concentrations are.  Figure 2 shows the values of the iFs. The highest values for the iFs are given by the population density and towns' geographical location where the meteorology area is significant for the dispersion and transportation of the pollutant. Factors of influence were similarly found; Tainio et al. [34] evaluated the iFs for the European population. They found that the iF value depends on the regional distribution of the population and the prevailing long-term meteorological conditions. Also, estimates varied 1.3 times when calculated by 5 to 30 kilometers domain resolution.

In total, 62.4% of the iFs in the domain are given in a radius less than 23 km and include the towns of Albania, Hatonuevo, and Barrancas which represent 44.3% of the total population of the area. Levy et al. [26] applied source-receptor (S-R) and CALPUFF matrix to seven power plants in northern Georgia to estimate iFs of fine particles; the results show that the 500 km radius receptor region captures approximately 70% of the total iFs in S-R matrix, but having used the CALPUFF model, we have estimated that approximately 50% of the total primary PM iFs are captured within 100 km. The fraction of total iFs can occur in the same area of emission with a variation ranging from 4% to 90%. Population size and the land area are related to the variation [34]. Marshall et al. [17] estimated that, for mobile source emissions, the contribution to population intake beyond 100 km is less than 2% due to a combination of low concentrations and low population density in surrounding areas. Heath et al. [37] assessed that the iFs within 100 km from each source capture 98% or more of the total iFs. Lamancusa et al. [38] appraised that a substantial fraction, >75% of the inhalation, occurs within 50 km, demonstrating that efforts to reduce emissions will have the largest health impact on the local community.

**Figure 3:**
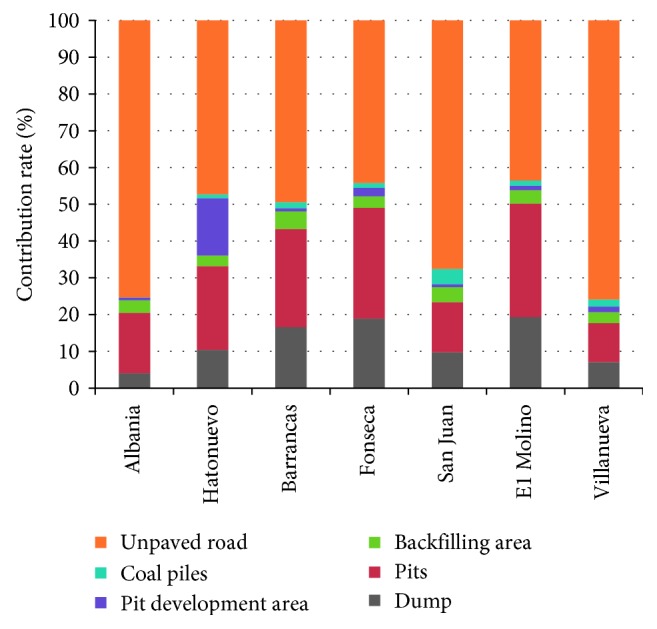
Annual average mining source contribution (%) among the PM_10_ intake fraction towns.

The contribution of the unpaved roads and pit emission has been important to the iF values (Figure 3). The contribution percentage to the iF values in towns, for unpaved road, varies from ranges between 43.55% (San Juan) and 75.35% (Albania). These values are significant; Mandal et al. [39] reported that the haul road contributes to 80.2% of the total dust generated during the operations of Indian opencast mines. Through material transportation, the haul/transport road becomes a major source of airborne particles thanks to road-tire interaction. Depending on air speed, particle diameter, and area of the material exposed to the atmosphere, direct emissions during transport can contribute to air pollution to long distances from sources. Previous investigations exhibited that unpaved roads have a concentration increase in the range source 952–1442 *µ*g·m^−3^, the highest among all mining activities [40]. According to the previous information, it can be inferred that a control on the unpaved road emissions has a direct impact on the public health indicators.

With the purpose of demonstrating the approximate magnitude of health impacts associated with these sources, we calculate an estimate point for morbidity impacts using standard epidemiological assumptions. We select a PM_10_ concentration-response function from the consequence of long-term exposure according to studies through time series [20, 21, 41–44]. We assume that all types of particles have toxicity equality and that the PM_10_ estimate concentrations are consistent with the PM_10_ environmental concentrations of the area according to the parameters of uncertainties estimated by the dispersion model.  Table 3 shows the annual health effects in each town due to opencast coal mine emissions in Northern Colombia. It is estimated that annually there are 22 hospital respiratory disease admissions, 442 emergency room visits, 105835 restricted activity days, and 336832 respiratory symptom cases attributable to the direct impact of the mining.

Opencast coal mine operations disperse a substantial amount of dust and other particles into the atmosphere which affects human health as a result of the increasing of local pollutants concentration into the atmosphere, such as PM_10_. Evidence indicates that people living in coal mining communities are at a great risk of developing heart and lung diseases like cancer, hypertension, and also kidney diseases; mortality rates are higher in communities located in close proximity to coal mines. Hendryx and Ahern [10] studied the relations between health indicators and residential proximity to coal mining through linear modeling; the results show that people, especially children, living within a radius of 1.6 km of an open-pit coal mine have an increase of 33% of having respiratory diseases, a cumulative increase of 21% to 3.2 km and up to 12% in less than 4.8 km. Other results show rates of cardiopulmonary and pulmonary diseases rise along with the coal production [11]. Brabin et al. [45] through ecological studies reveal the incidence of respiratory symptoms in schoolchildren in areas exposed to coal dust in a 2 km radius, and the respiratory symptoms were more frequent including wheezing, coughing in excess, and school absences due to respiratory diseases. Our study assists as a screening test to examine whether coal mining poses a health risk for people that inhale pollutants caused by those emissions. Confirmatory tests should be undertaken to establish mechanisms of action, magnitude, and health consequences of the exposure.

This analysis uses air dispersion models to assess the concentrations at which population near to an identified mining source is exposed. Therefore, there is uncertainty associated with the dispersion model. The CALPUFF model was selected to carry out this study because of its suitability for the type of terrain and the treatment of the sources. On the other hand, model inputs include emission rates, emission release parameters, output conditions of the Calumet weather model, and dispersion coefficients. Each of the inputs to the dispersion model has an associated uncertainty. Among these inputs, emission rates and meteorological conditions have the greatest effect on the model results. A major limitation of the study is the underestimation of PM_10_ concentrations near the sources. Within a model grid cell, the pollutant concentration and the population are assumed to be evenly distributed, that is, to be spatially noncorrelated. In reality, this is clearly not true. The underestimation of exposures near the emission sources leads to underestimation of the intake fractions. We assumed that the population remains static in their place of residence over time. In reality, people go to work, travel, and do different activities, and this often takes place at times when the mine emissions are high during sun hours. In addition, there are uncertainties associated with health values derived from the potential risk for the general public because there is a wide range of responses between all individuals and the actual exposure time to the emitted concentrations. Risk assessment is a complex process that requires integration of many variables and assumptions. As a result of these variables and assumptions, there are uncertainties and limitations for the results. However, the results are approximations to the reality that allows us to understand different process impacts.

The use of this general approach for cohort morbidity benefit estimation has been criticized; these models are useful to assess impacts due to changes in long-term exposure. The use of contamination models and employment of transverse ecological studies is becoming more important due to the inference and the approximations that consent to value them based on the existing database. Those economically versatile and feasible investigations allow selecting new hypotheses for deeper studies on the pollution effects for human health. Formulations found in this research show the significant effect of PM_10_ emissions from mining sources in the observation area. It appears that 1 kilometer is inadequate to determine substantial values of intake fraction at the population, emphasizing the importance of accurate long-range dispersion modeling. A formal structured uncertainty analysis or value of information analysis would be needed to provide better quantitative evidence of the relative importance of different components of an opencast coal mine risk assessment. Future research efforts should address such needs, by supplementing these comparisons with calculations from other atmospheric models (Aermod and Austal 2000 model) and other regions and evaluating within-model and between-model uncertainties for other mining source categories. It is advisable for mining companies to improve the dispersion model as a tool for greater management of mining emissions. Also, it is suggested to perform a quasiexperimental epidemiological study to have a better understanding of mining impacts on public health in the exposed areas.

## 4. Conclusions

In this study, we estimated PM_10_ intake fractions for an opencast coal mine in Northern Colombia. Variation of iF values have been found in the order of 10^−8^ for towns located in domain; these are mainly substantial due to the geographic location in terms of the spatial distribution of populations and that it may represent in context of public health. We appraised that annually there are 22 hospital respiratory disease admissions, 442 emergency room visits, 105835 restricted activity days, and 336832 respiratory symptom cases attributable to the direct impact of the mining. The significant portion of iFs occurs beyond the source, emphasizing the need to use more finite computational spatial resolution in future studies in order to avoid underprediction of caused exposure. Although this analysis does not yield to a definitive model validation or uncertainty estimates, this approach provides meaningful insight for the importance of underlying variables for assessing population about exposure and risks. This information can help in setting priorities among competing control strategies for pollution control in Colombia, such as setting regional priorities in pollution control, and conducting policy simulations.

## Figures and Tables

**Table 1:** Open-pit coal mine PM_10_ emissions for 2014. Dump, pit, backfilling, pit development, coal piles, and unpaved road are the area sources considered within mine (g·m^−2^·s^−1^).

	Dumps	Pits	Backfilling area	Pit development area	Coal piles	Unpaved road (g·s^−1^)
Northern zone	5.08 × 10^−6^	1.65 × 10^−5^	1.34 × 10^−5^	3.76 × 10^−6^		85.23
Central zone	1.40 × 10^−6^	6.14 × 10^−6^	3.37 × 10^−7^	3.07 × 10^−6^	3.60 × 10^−5^	29.04
South zone	1.14 × 10^−5^	3.10 × 10^−5^	2.05 × 10^−7^	1.69 × 10^−5^		19.86
All	1.79 × 10^−5^	5.37 × 10^−5^	1.39 × 10^−5^	2.37 × 10^−5^	3.60 × 10^−5^	134.13

**Table 2:** Mean, standard deviation, coefficient variation, and maximum intake fractions for towns located in Northern Colombia.

	Mean	Standard deviation (SD)	Coefficient variation (CV)	Maximum
Albania	3.53 × 10^−8^	7.17 × 10^−8^	2.03	3.77 × 10^−7^
Hatonuevo	1.25 × 10^−8^	2.16 × 10^−8^	1.73	9.45 × 10^−8^
Barrancas	3.66 × 10^−8^	2.14 × 10^−8^	5.83 × 10^−1^	8.58 × 10^−8^
Fonseca	2.35 × 10^−8^	1.23 × 10^−8^	5.23 × 10^−1^	4.83 × 10^−8^
San Juan	6.13 × 10^−9^	4.55 × 10^−9^	7.42 × 10^−1^	1.49 × 10^−8^
El Molino	9.69 × 10^−9^	5.48 × 10^−9^	5.65 × 10^−1^	1.99 × 10^−8^
Villanueva	1.15 × 10^−8^	6.29 × 10^−9^	5.47 × 10^−1^	2.40 × 10^−8^

**Table 3:** Annual health effect assessment data for intake fraction.

Towns	Hospital respiratory admissions	Emergency room visits	Restricted activity days	Respiratory symptoms
Albania	2	48	11580	36856
Hatonuevo	1	12	2767	8806
Barrancas	8	165	39637	126148
Fonseca	7	133	31956	101704
San Juan	2	45	10820	34435
El Molino	0	7	1600	5093
Villanueva	2	31	7475	23790

## Data Availability

The data used to support the findings of this study are available from the corresponding author upon request.

## Additional Points

*Highlights*. (i) Opencast coal mine emits PM_10_ at a high rate into the atmosphere. (ii) The impact of opencast coal mine is on a large scale. (iii) The biggest health impact on the population near mining sources is on the respiratory system.
